# Thymoquinone attenuates cisplatin-induced hepatotoxicity via nuclear factor kappa- β

**DOI:** 10.1186/1472-6882-14-282

**Published:** 2014-08-03

**Authors:** Abdulrahman L Al-Malki, Ahmed Amir Radwan Sayed

**Affiliations:** Department of Biochemistry, Faculty of Science, King Abdulaziz University, P.O Box 80203, Jeddah, Saudi Arabia; Chemistry Department, Faculty of Science, Minia University, El- Minia, Egypt

**Keywords:** *Nigella Sativa*, NF-κB, CP, hepatotoxicity, TNFα, IL-1β

## Abstract

**Background:**

Cisplatin (CP) is known as a potent anti-cancer drug. The most therapeutic adverse effect of CP is induced hepatotoxicity. In the present study, the protective effect of thymoquinone (TQ) on CP-induced hepatotoxicity was studied.

**Methods:**

Wistar rats were divided into three groups (15 rats each). Group 1 served as the control group. Group 2 rats were injected *ip* with a single dose of CP (12 mg/kg b.w, *i.p.*). Group 3 rats were orally pre-treated with TQ (500 mg. kg^−1^. day^−1^) for one month, then the animals were injected *i.p* with CP 12 mg.kg^−1^.

**Results:**

The beneficial effects of TQ with its antioxidant/anti-inflammatory effects were observed. Injection of rats with CP markedly affected the liver functions and histopathological changes. The antioxidant enzyme activities and reduced glutathione (GSH) contents were significantly decreased while the levels of malondialdehyde (MDA) significantly increased. The electromobility shift assay (EMSA) showed a significant activation of NF-κB-p65 in the rat liver injected with CP. Furthermore, the expression and concentrations of inflammatory tumor necrosis factor (TNF-α), nitric oxide synthetase (iNOS), and interleukin (IL-1β) were markedly elevated in the CP injected rats. The administration of TQ improved all the altered functions, histopathology of the liver and attenuated the activated NF-κB. The antioxidant enzyme activities (glutathione peroxidase and glutathione –S transferase) of the rat livers were markedly increased while MDA was reduced as a result of TQ administration. In addition, the expression of TNF-α, iNOS, and IL-1β were markedly reduced.

**Conclusion:**

It was concluded that, TQ has potential benefits in the prevention of the onset and progression of CP induced hepatotoxicity.

## Background

The modern life style and environmental pollution, have been the causes of increased cancer burdens in the world. Chemotherapy is one of the most important methods used in cancer therapy. CP [cis-diamminedichloroplatinum (II)] (CP) is a well known anticancer drug. It is primarily used as a drug in the treatment of solid tumors. Use of CP in the treatment of tumors is restricted due to its toxic effect on kidney and liver, which can be seen after a single dose of CP in approximately 28% to 36% of cancer patients [1]. CP is a small molecule which can easily cross the plasma membrane and then to nucleus. In the nucleus, CP causes changes in the structure of the DNA molecule. These changes result from the formation of inter- and intra-chain adducts between CP and the nitrogen bases of the DNA [2]. Oxidative stress plays the key role in the CP induced hepatotoxicity. Previous studies showed that, the earliest signs of CP induced hepato-toxicity are the fall in the hepatic reduced glutathione (GSH) levels and an increase in the hepatic malondialdehyde (MDA) levels [3]. These signs indicate the acceleration of the peroxidative processes in the hepatic cell [4, 5].

The oxidative stress and production of reactive oxygen species (ROS) such as hydroxyl radicals, superoxide anions, and hydrogen peroxide are normally generated in liver. A detoxification mechanism working in the liver detoxifies the ROS by endogenous antioxidants such as GSH, SOD, and catalase. The accumulation of intracellular ROS leads to an increase in both DNA damage and peroxidation of membrane lipids [4].

The exogenous production of free radicals may originate from different factors such as pollution of water and air and radiation exposure. Further, endogenous free radical production results from normal metabolism. Many chronic diseases, like cancer, diabetes, Alzheimer, and cardiovascular diseases are associated with free radical production. Free radicals are highly reactive intermediates. They react with nucleotides of DNA, the sulphhydryl bonds of protein, and the polyunsaturated fatty acids found in cellular membranes that result in tissue damage [6]. Prevention and/or decreasing of the side effects of CP are of the main concerns in treating patients suffering from cancer. Different methods were established to attenuate the toxic effects of CP, such as combination of the free radical scavengers [7] but it is not efficient.

Thymoquinone (TQ) is a phytochemical that has been reported to exhibit antimicrobial effects against different species of bacteria. TQ is considered as potent anti-oxidant, anti-carcinogenic and anti-mutagenic agent. Moreover, TQ is a relatively safe compound, particularly when given orally to experimental animals [8].

TQ is a quinone isolated from *Nigella sativa* seeds. Previous study showed that TQ has a significant protective action toward an array of free radical creating compounds like doxorubicin [9]. In addition TQ has an anti-inflammatory as well as an analgesic action and protects against carcinogenesis induced by different chemical compounds [10]. Moreover, TQ has protective effect against both diabetic nephropathy [11–13] and membrane induced lipid peroxidation [8]. Furthermore, it has been showed that TQ is a potent suppressor of iron-NTA induced oxidative stress and carcinogenesis in the rat kidney [14].

The aim of the present study is to evaluate the protective effects of TQ on the CP-induced hepatotoxicity in rats. In addition, the mechanism of action of TQ was studied to show the impact of oxidative stress as well as the antioxidants in the development and attenuation of CP-induced hepatotoxicity.

## Methods

### Chemicals

Thymoqunione (TQ) was purchased from Sigma-Aldrich (St. Louis, MO, USA). BCA mega kit (Pierce, Rockford, USA). All assay kits were obtained from BIORAD (England). Primers were obtained from Bioline Inc., (Taunton, MA, USA). Monoclonal antibody, anti- NFkBp were obtained from Bioline Inc. All the chemicals used in the study were of analytical grade.

### Animals

Male Wister rats weighting 190 ± 30 g were kept for 7 days with free access to water ad libitum and standard rat chow.

All the animal protocol was approved from the University Animal Ethics committee. CEGMR committee licensee # at KASCT. HA-02 J-003.

Rats were randomly divided into three groups each consists of 15 rats. Group 1 served as the normal control group. Animals of this group were treated with normal saline intrapertoneal (ip). Group 2 was injected *i.p.* with a single dose of CP (12 mg/kg b.w, *i.p.*) obtained from Sigma (England). Group 3 was orally pre-treated with TQ in polypropylene glycol (500 mg. kg^−1^. day^−1^) [12] for one month, then the animals were injected *i.p.* with CP 12 mg.kg^−1^ body weight [15]. TQ treatment was continued for one month. At the end of the experiment, rats were sacrificed, the blood was collected, and serum was separated and stored at −80°C. Serum was used for the determination of serum lactate dehydrogenase (LDH), alanine amino transferase (ALT), γGGT, aspartate amino transferase (AST), total protein (t. Protein) alkaline phosphatase (ALP), albumin, and total bilirubin.

Liver was isolated and rinsed carefully with cold normal saline and divided into four parts. The first part was used to prepare the tissue homogenate in phosphate buffered saline (50 mmol/l, pH 7). The liver homogenate was used for the assay of the activity of superoxide dismutase (SOD), catalase, glutathione peroxidase (GSH-Px), glutathione S transferase (GST), and the levels of reduced glutathione (GSH), malondialdehyde (MDA), NO, IL-1β and TNFα. The second part of the liver was used in the preparation of the tissue extract for electromobility shift assay (EMSA) analysis. Total RNA was extracted from the third part of the liver and the fourth part was used for histopathological examinations.

### Assay of ALT, AST, ALP, t. protein, albumin, t. bilirubin, and γGGT

ALT, ALP, AST, albumin, t. protein, γGGT and t. bilirubin in the serum of all rats were assayed using the available kits obtained from Bio Vision Research Products (Avenue, USA). All assays were made according to the instructions of the manufacturer.

### Determination of LDH

The method of King [16] was used for the assay of LDH using kit from BIORAD (England).

#### Assay of reduced GSH

Reduced GSH was assayed by the colorimetric end point method as described by Sayed [17] using kit from BIORAD (England).

#### Determination of the activity of glutathione peroxidase

The method of Paglia and Valentine [18] was used for the determination of the activity of *glutathione peroxidase* in the liver homogenate using kit from BIORAD (England). The activity was expressed as U/mg protein.

#### Assay of GST activity

The activity of GST was determined as described by Moron et al*.* [19] by using 1-chloro −2, 4- dinitro benzene (CDNB) as an electrophilic substrate using kit from BIORAD (England). The activity was expressed as nmol/mg protein.

#### Assay of malondialdehyde (MDA)

Malondialdehyde (MDA) was assayed using the method described [20, 21]. The concentration of MDA was calculated and expressed as nmol . mg^−1^ protein.

#### Assay of the activity of SOD

The activity of super oxide dismutase was assayed as described [22] using kit from BIORAD (England). The activity was expressed as U/mg protein.

#### Assay of the nitric oxide concentration

The formation of NO was determined by indirect method by quantifying the tissue level of nitrite by a calorimetric method by Griess reaction [23] using kit from BIORAD (England).

### Assay of IL-1β and TNF-α

*IL-1β* and TNF-α levels were assayed using an available ELISA kit from R & D (Berlin, Germany) according to the manufacturer’s instructions [24]. Protein concentration in the liver homogenate was assayed using BCA mega kit (Pierce, Rockford, USA).

### RT-PCR

The transcripts were amplified in a single reaction containing 1 mg cDNA and 0.5 mM each of the sense and antisense primers for IL-1β ( sense 5′- AT GG CA AC TG TC CC TG AA CT C -3′; antisense 5′- GT CG TT GC TT GT CT CT CC TTG -3′ ), TNFα sense 5′- TCATGCACCACCATCAAGGA-3′ and antisense 5′- GA CA TT CG AG GC TC CA GT GA A -3′ and iNOS sense 5′-CA GC CA AG TA TT CC AA AG CA A −3′ and antisense 5′- AG TC CA GT CC CC TC AC CA A −3′; *β- actin* sens 5′ GT GC TA TG TT GC TC TA GA CT TC G −3′, antisens 5′ AT GC CA CA GG AT TC CA TA CC −3′ were obtained from BIOLINE Inc., (Taunton, MA, USA).

The cycle consistes of preheating step at 95°C for 5 minute followed by 35 cycles of 90°C for 1 minute, and 60°C for 30 seconds.

### EMSA analysis

Part of the liver was dissolved in TOTEX buffer. The extract was used for determination of the binding activity using NF-κBp65 consensus sequence: 5′-AG TT GA GG GG AC TT TC CC AG GC -3′ which was obtained from BIOLINE Inc. (Taunton, MA, USA). The increase in the expression of NFkBp65 gene was determined by a Phosphor Imager using background subtraction [25].

### Histopathological examination

Liver tissues were collected from rats, washed carefully by cold normal saline 3 times, then fixed in formalin solution 10%, processed, and embedded in a paraffin film. Sections of 5-μm thick slices of liver were prepared. The sections were stained with H & E. The method was described in Fukuzawa et al. [26].

### Immunohistochemistry

The sections of tissues in phosphate-buffered saline (pH = 7.2) were incubated overnight at 4°C with the respective primary monoclonal antibody, and anti- NFkBp dilutions (1:100, 1:50). Immunohistochemical staining was performed by the streptavidin-biotin complex method. All sections were then counterstained with hematoxylin.

### Statistical analysis

All values are expressed as the mean ± SD. Data were evaluated by using SPSS for windows. The one way ANOVA test was used to examine whether there are any significant differences between the treatment groups, and the value of *P* < 0.05 was considered significant.

## Results

### Initial and final body weights

Both the initial and the final body weights of all rats are presented in Table 1. There were no significant differences in the initial body weights among all three groups. Rats treated with CP (group 2) showed a significant decrease in the final body weights (group 2) when compared to the control group. As a result of TQ treatment, the rats of group 3 showed a significant increase in the final body.

**Table 1:** **Biochemical parameters of liver function tests as well as initial and final body weights of rat**

	Group 1	Group 2	Group 3
Initial body weight, g	210 ± 10	203 ± 12	201 ± 12
Final body weight, g	243 ± 18	172 ± 12^a^	225 ± 12^b^
ALT, U/L	29.12 ± 1.12	98.32 ± 2.3^a^	39.2 ± 1.3
AST, U/L	26.78 ± 1.5	90.78 ± 1.14^a^	48.5 ± 5.3
ALP, U/L	167.34 ± 2.32	369.12 ± 18.8^a^	215.3 ± 11.2^b^
LDH, U/L	75.67 ± 2.12	155.56 ± 2.4^a^	112 ± 4.2^b^
γGGT, U/L	59.36 ± 1.45	109.5 ± 2.43^a^	85 ± 3.5
t. protein, mg%	9.53 ± 0.76	6.15 ± 1.27	6.8 ± 2.1
Albumin, mg%	3.94 ± 0.98	2.4 ± 0.54^a^	4.65 ± 2.5^b^
t. Bilirubin, mg%	0.75 ± 0.05	1.6 ± 0.23^a^	1.1 ± 0.01^b^

Values are expressed as mean ± S.D. of triplicate tests (n = 15).

^a^ *P* < 0.05 vs group1, ^b^ *P* < 0.05 vs group 2.

The effect of TQ on the liver function parameters of different groups.

### Effect of CP and TQ administration on the hepatic biomarkers

Table 1 shows that the injection of CP significantly increased the hepatic levels of ALT, ALP, AST, γGGT, total bilirubin and LDH and a significant decrease of serum albumin when compared to the control group. Administration of TQ significantly improved the negative effects of some of the hepatic biomarkers and modulated the other toxic effects.

### Effect of TQ administration on glutathione peroxidase activity

As a result of CP treatment, the activity of GSH-px in the liver tissue of the rats in group 2 was significantly reduced when compared with the control group (group 1) (Table 2). On the other hand, treatment of rats in group 3 with TQ resulted in a marked increase of GSH-px activity when compared to the CP treated rats (group 2) (*P* < 0.05).

**Table 2:** **Antioxidant parameters of rat liver**

	Group 1	Group 2	Group 3
MDA, nmol/mg protein	0.52 ± 0.06	5.1 ± 0.85^a^	0.95 ± 0.02^b^
GSH, nmol/mg protein	111 ± 5.8	55.9 ± 4.6^a^	89.5 ± 2.1^b^
GST, nmol/mg protein	18.6 ± 1.5	6.29 ± 0.39^a^	14.76 ± 0.86^b^
SOD, U/mg protein	15.8 ± 1.1	9.6 ± 2.1^a^	14.25 ± 1.1^b^
CAT, U/mg protein	5.95 ± 0.5	2.41 ± 0.31^a^	4.56 ± 0.54^b^
GSH-Px, U/mg protein	4.95 ± 0.21	2.05 ± 0.12^a^	4.67 ± 0.65^b^

The expressed values are mean ± S.D. of triplicate tests (n = 15).

^a^ *P* < 0.05 vs group1, ^b^ *P* < 0.05 vs group 2.

The activity of the antioxidant enzymes as well as the levels of MDA and GSH in the liver homogenates of different groups.

### Effect of TQ administration on superoxide dismutase activity

SOD plays an important role in detoxification of the hydroxyl radicals. The activity of SOD in the liver homogenates of all experimental rats is shown in Table 2. In group 2, treatment of rats with CP reduced the SOD activity compared to the control group. Enhancement of SOD activity was observed as a result of TQ treatment, and the activity was found to be markedly increased.

### Effect of administration of TQ on glutathione-S-transferase activity

Table 2 shows a significant decrease in the hepatic GST activity upon CP treatment as compared to the control group (P < 0.05). A significant increase in GST activity was observed in the rat group treated with TQ as compared to the CP treated group (*P* < 0.05).

### Effect of TQ administration on glutathione levels

CP treatment significantly decreased GSH level in the liver tissue homogenates compared to the control group. Rats treated with CP had a lower GSH content than the normal control group. Administration of TQ significantly modulated these alterations and the level of GSH rose (P < 0.05) as shown in Table 2.

### Effect of administration of TQ on lipid peroxidation

Formation of free radicals and induction of oxidative stress is a direct cause of lipid peroxidation. Lipid peroxidation was measured as MDA in rat liver homogenate and the results are shown in Table 2. After 12 mg/kg CP injection, the liver MDA level significantly increased. Administration of TQ significantly decreased the amount of MDA in the CP treated rats of group 3. The oral administration of TQ decreased the level of MDA.

### Effect administration of TQ on catalase activity

As a result of CP injection, catalase activity was markedly reduced. Table 2 shows that the activity of catalase was reduced in the CP treated group. However, the oral administration of TQ increased the activities.

### Effect of CP and TQ treatment on NO, TNFα and IL-1β

**Figure 1:**
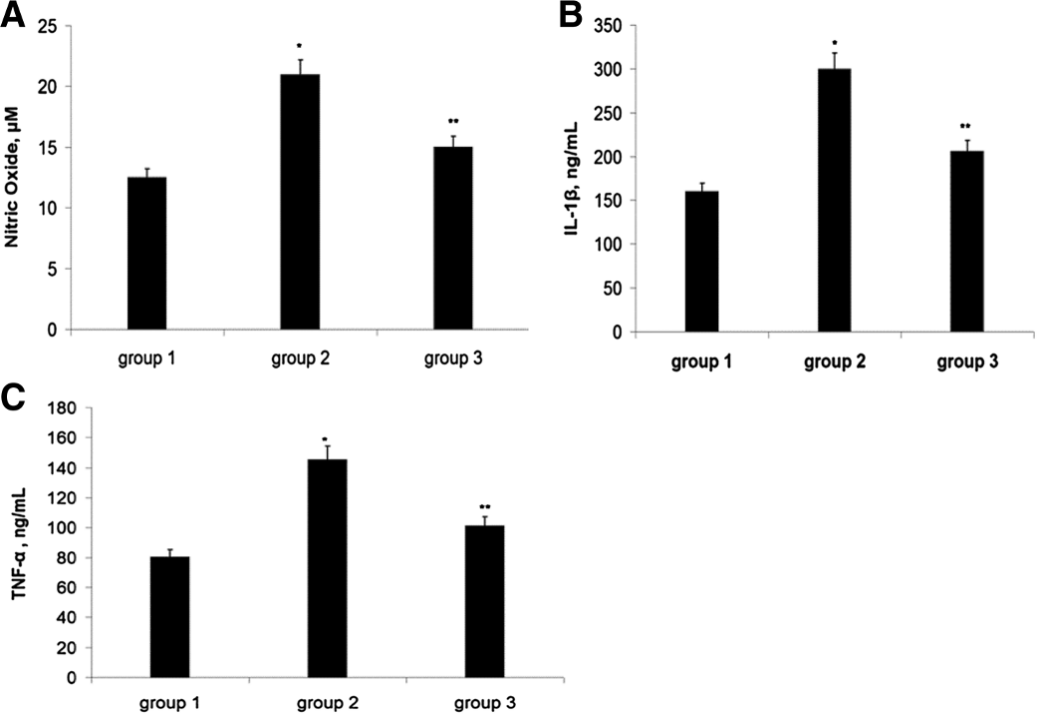
**Effect of TQ on the levels of nitric oxide (A), IL1-β (B), and TNFα (C) of CP treated rats.** Results are presented as mean ± SD, *P < 0.05 against group 1, **P < 0.05 against group 2.

The effect of CP as well as the effect of TQ on NO, TNFα and IL-1β in rats was studied. Figure 1 shows that the level of NO was markedly and dose dependently increased as a result of CP treatment. Administration of TQ reversed these effects and significantly reduced the levels of NO in the treatment group 3 (Figure 1A).

The levels of TNFα and IL-1β were significantly and dose dependently elevated in the CP treated rats (group 2) as indicated in Figure 1B and C. The elevated levels of TNFα and IL-1β were significantly reduced as a result of TQ administration.

**Figure 2:**
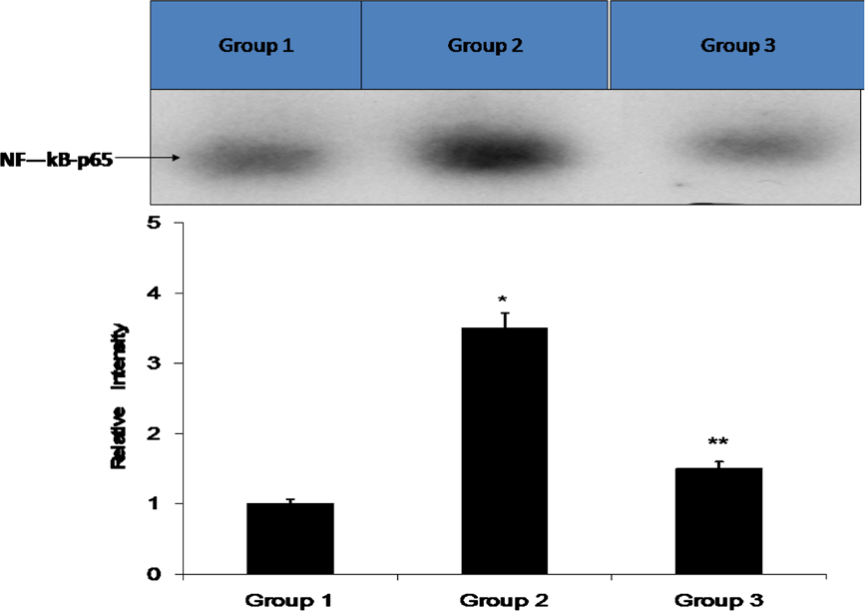
**Binding activity of NF- κB.** Quantification of activated NF-kB was performed by densitometric analysis of relative EMSA band intensities. Results are the means ± SE of four individual replicates; value from an unpaired Student’s *t*-test (*P < 0.05, compared with group 1 and **P < 0.001) comparing with group 2.

NF-κB-p65 was markedly activated in the CP treated animals compared with the control. Administration of TQ resulted in a significant reduction in the of NF-κB-p65 in a dose dependent manner (Figure 2).

**Figure 3:**
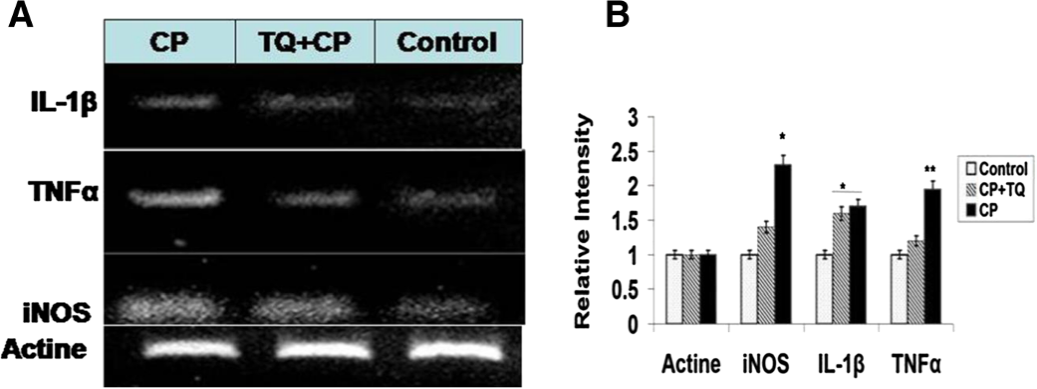
**Effect of CP on IL1-β, TNF-α and iNOS expression (A).** The relative intensity of the bands is represented in **(B)** Data are the mean ± S.D. (n15); *P < 0.01, **P < 0.01 vs. control animals.

As a result of oxidative stress, NF-κB-p65 was markedly activated in the cisplatin treated animals compared with the normal control. Administration of TQ resulted in a significant reduction of the of NF-κB-p65 activation. The levels of TNFα and IL-1β were significantly and dose dependently elevated in the CP treated rats (group 2) as indicated in Figure 1B and C. The elevated levels of TNFα and IL-1β were significantly reduced as a result of TQ administration (Figure 3).

**Figure 4:**
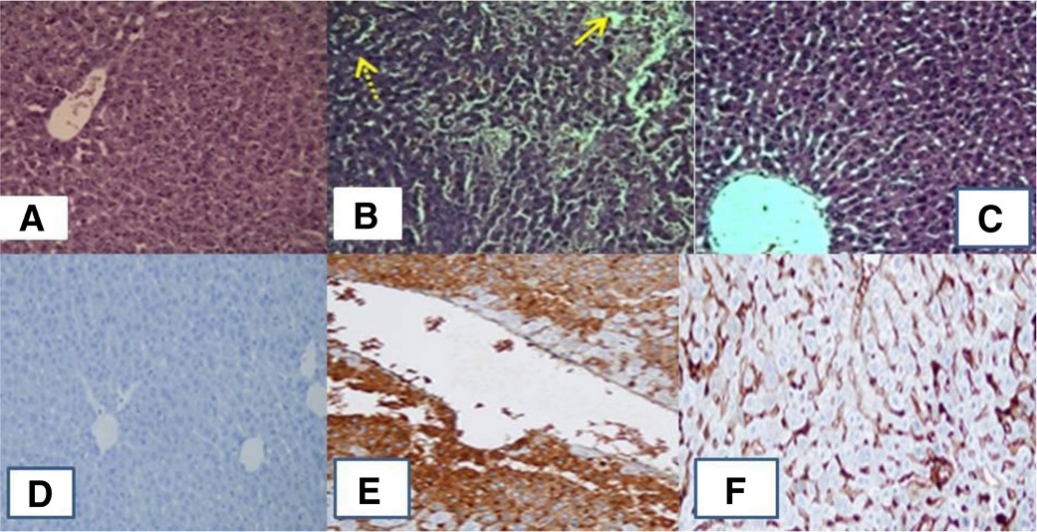
**Histopathological and immunohistochemical observation of rat liver sections Vacuolar degeneration (dotted arrows) and hepatocellular necrosis (complete arrows) are displayed). (A)**: Control group; **(B)**: group treated 12 mg/kg cisplatin; **(C)**: group pretreated with thymoquinone then cisplatin as discussed in methods. Immunohistochemical staining of NF-κB in rat from: (**D**, 200) group 1, showing no expression of NF-κB; (**E**, 200) group 2 showing a significant increase in NF-κB immunoreactivity in the cytoplasm of hepatocytes; and (**F**, 200) group 3 demonstrating a significant reduction in NF-κB immunostaining.

Rats which received cisplatin showed a significant increase in the immunoreactivity of NF-κB in the cytoplasm of hepatocytes compared with the normal control group. On the other hand, thymoquinone-treated rats showed considerable reduction in the cisplatin-induced expression of NF-κB in the liver compared with the cisplatin treated rats group 2 (Figure 4D-F).

## Discussion

Thymoquinone (TQ) showed diverse therapeutic benefits as related to bronchial asthma, dysentery, headache, gastrointestinal problems, eczema, hypertension, and obesity, antiproliferative effects on cell lines derived from breast, colon, ovarian, larynx, lung, myeloblastic leukemia, and osteosarcoma. Mechanistically, TQ induced apoptosis in tumor cells by suppressing NF-κB, Akt activation, and extracellular signal-regulated kinase signaling pathways and also suppress tumor angiogenesis. These studies suggested that TQ is useful as conventional chemotherapeutics against cisplatin induced cytotoxicity. It was found that, TQ was effective as inducer of apoptosis in some types of cancer by down-regulating several antiapoptotic proteins such as Bcl-xL [9].

The hepatic cell contains high levels of transaminases as AST. As a result of the damage caused to hepatic cells by CP, the leakage of cytosol resulted in increasing levels of the liver specific enzymes in the rat serum. The elevated levels of the hepatic enzymes in the serum such as AST and ALT are markers that indicate the cellular leakage and the functional integrity of the liver cell membrane [27]. The serum levels of ALT and AST serve as a indirect assessment of the condition of the liver. In the present study, the capability of TQ in ameliorating the CP-induced hepatotoxicity was observed in that the rats of group 3 had lower levels of the hepatic enzyme in the serum when compared with the animals of group 2.

TNF-α is a pro-inflammatory cytokine. Its levels are elevated in the acute and chronic disease conditions. The anti-inflammatory action of TQ was estimated *in vivo* by measuring the release of TNF-α. Previous studies showed that natural products have the ability to reduce inflammation by blocking the inflammatory mechanisms downstream of the release of cytokines, and also by attenuating the production of the proinflammatory factors by macrophage [28]. It was reported that the proinflammatory cytokines like TNF-α play an important role in the pathogenesis of a large number of liver diseases. TNF-α is released from the activated Kupffer cells. Following its release, it aggravates both the inflammatory response and oxidative stress in the hepatic cells [29]. In addition, it induces the production and release of chemokines and cytokines from macrophages and stimulates phagocyte nitric oxide (NO) production and oxidative metabolism. Although previous studies showed that the production of NO protects the liver from injury by a NOS inhibitor, recent data shows that the excessive production of nitric oxide by iNOS can result in hepatic injury [30]. A recent report showed that the overproduction of iNOS in the rat liver is related in the acute liver injury. This suggested that iNOS could act as a mediator in the pathogenesis of the liver toxicity in rats [31].

In this study, we showed that the liver protective effect of TQ was not only attributed to its inhibitory effect on the release of the inflammatory mediators NO, TNF-α, and IL-1β, but also to its antioxidant properties. MDA, a product of lipid peroxidation, was increased in the rat liver as a result of CP injection. However, we showed that TQ significantly reduced MDA formation. In other words, the mechanism of the inhibitory effects, by which TQ protects against lipid peroxidation, may involve radical scavenging capability. Hepatotoxicity not only initiates lipid peroxidation but also reduces tissue GSH-px, GST, CAT, and SOD activities, and this depletion may result from oxidative modification of these proteins and our data are in line with other previous studies [32–36].

NF-κB is a nuclear transcription factor found in the cytoplasm of the liver cells. In the cytoplasm, it binds to its inhibitory subunit I-κB. As a result of oxidative stress, NFκB is activated and phosphorylated from its inhibitory subunit. As a result of its activation, NF-κB transfers to the nucleus of the hepatic cell, binds to DNA and up-regulates the transcription of many inflammatory genes like cytokine, chemokine and receptors of advanced glycation end products [31]. Our data from the EMSA and immunohistochemistry showed a marked activation of NF-κB which are in line with the hypothesis above. In addition the data on the levels of TNF-α, NO and IL-1β and the expression of the TNF-α, iNOS and IL-1β genes support the results of the EMSA and immunohistochemistry. Our study is in accordance with the study that reported that TQ induced apoptosis in tumor cells by suppressing NF-κB, Akt activation, and extracellular signal-regulated kinase signaling pathways and suppressing tumor angiogenesis. These studies suggested that TQ is useful as conventional chemotherapeutics against cisplatin induced cytotoxicity. It was found that TQ was effective as inducer of apoptosis in some types of cancer by down-regulating several antiapoptotic proteins such as Bcl-xL. For this reason black Nigella seeds is widely used in baking of bread and other foods, as additive for spiced and flavored and aromatic substances. Nigella seeds are reported as antifungal, antibacterial, antiviral and antihelmintic, treatment of flatulence and abdominal ailments, decrease fasting plasma glucose concentration in rabbit, increase serum total protein, as diuretic, hepatoprotective and hypotensive.

The present study clearly demonstrates that controlling of oxidative stress and catalytic reactive oxygen scavenging are effective in the prevention of CP induced hepatotoxicity. TQ is the major constituent of the natural food supplement (*Nigella sativa*) with a broad spectrum of beneficial biochemical and cellular biological effects, based on its ability to reduce inflammation and ROS overproduction. Since TQ also has beneficial effects on other target tissues and shows beneficial effects of mediators of large vessel damage, this mechanism appears attractive for the prevention or delay of many disorders.

## Conclusion

Our findings show that TQ has the potential benefits in the prevention of the onset and progression of CP induced hepatotoxicity.
